# Psychotic mania as the solitary manifestation of neurosyphilis

**DOI:** 10.1186/s12991-018-0195-1

**Published:** 2018-06-06

**Authors:** Eun Hyun Seo, Hae Jung Yang, Sang Hoon Kim, Jung Hyun Park, Hyung-Jun Yoon

**Affiliations:** 1 Premedical Science, College of Medicine, Chosun University, Gwangju, Republic of Korea; 2 Department of Psychiatry, Chosun University Hospital, Chosun University, Gwangju, Republic of Korea; 3 Department of Psychiatry, College of Medicine, Chosun University, Gwangju, Republic of Korea

**Keywords:** Neurosyphilis, Bipolar disorder, Psychiatric manifestation, Blonanserin

## Abstract

**Background:**

Neurosyphilis remains a diagnostic challenge in current psychiatric practice because of its pleomorphic psychiatric manifestations. Although neurosyphilis can present with a wide range of psychiatric symptoms, psychotic mania as its solitary manifestation is an unusual phenomenon.

**Case presentation:**

A 46-year-old man, with no history of any psychiatric disorder, exhibited abruptly developed symptoms of psychotic mania. He was admitted to a psychiatric ward for further evaluation and treatment. Upon admission, his cognitive function was unimpaired, and the hyperactivity was not severe. Also, no abnormalities were found upon neurological examination and brain magnetic resonance imaging. He was initially diagnosed as bipolar disorder with psychotic features. On the 3rd day after admission, he was confirmed as having neurosyphilis by analysis of cerebrospinal fluid and treated with intravenous penicillin—in combination with blonanserin—an atypical antipsychotic drug. After 2 weeks of treatment, most of the symptoms had abated.

**Conclusions:**

The present case emphasizes that patients presenting with atypical psychiatric manifestation should be screened for possible syphilis, particularly in the absence of previous psychiatric history, and suggests that combination of blonanserin with antibiotic therapy may be effective in the treatment of the manic and psychotic symptoms secondary to neurosyphilis.

## Background

Although the prevalence of syphilis remarkably decreased after widespread use of penicillin, there appears to be a resurgence of syphilis in the United States and several European countries [1]. The reemerging syphilis has been found to occur predominantly in high-risk groups, such as those infected with human immunodeficiency virus (HIV) [2]. Neurosyphilis is caused by the invasion of *Treponema pallidum* into the central nervous system, which can occur at any stage of syphilis and lead to severe neurological sequelae [3]. Since its remission to treatment depends on the duration of illness, early detection and treatment are critical [4]. Nevertheless, syphilis is usually no longer considered a differential diagnosis for new onset psychiatric symptoms, and is not routinely screened for, unless patients seem to be in particularly high-risk groups. Often referred to as the “great imitator,” neurosyphilis can present with various psychiatric symptoms, including psychosis, personality change, delirium, and dementia [5]. However, psychotic mania as the solitary manifestation of neurosyphilis remains a rare phenomenon. Here, we report such a case of neurosyphilis which was initially diagnosed as bipolar disorder.

## Case presentation

A 46-year-old unmarried man was referred to the Department of Psychiatry, at Chosun University hospital by a local psychiatric clinic. Acute euphoric mood, labile affect, increased talkativeness, decreased need for sleep, and unrestrained buying sprees had developed about 3 weeks prior. Concurrently, he was convinced that he had sufficient money to start a global business, and was scheduled to meet the President of his country in secret. In addition, he experienced vivid visual hallucinations: he reported having recently seen hundreds of North Korean soldiers in the battlefield at dawn. He had already been prescribed psychotropic medications, including aripiprazole (5 mg/day) and alprazolam (0.5 mg/day), at a local psychiatric clinic 2 days prior. He was admitted to a psychiatric inpatient unit for further evaluation and treatment. Upon admission, the patient was found to have no personal or family history of psychiatric illnesses. He reported no sexual intercourse over the last 3 years. On mental status examination, he showed the characteristic psychopathology of psychotic mania, such as euphoric mood, grandiose delusions, flight of ideas, pressure of speech, and increased psychomotor activity. However, he did not show any impairment in concentration and immediate memory. His Mini-Mental State examination (MMSE) score was 29; he only failed to recall one item at delayed recall, reflecting unimpaired cognition. Further, no abnormalities were found upon physical and neurological examination. He was diagnosed with bipolar disorder with psychotic features. On the 1st day after admission, blonanserin, an atypical antipsychotic drug, was administered at a dose of 8 mg/day for alleviation of manic and psychotic symptoms.

On the 2nd day, routine blood test, including complete blood count, serum electrolyte, and biochemical tests, and chest X-ray, electrocardiography, electroencephalography, and brain magnetic resonance imaging (MRI) showed no aberrant findings. However, the patient showed a positive result for the serum venereal disease research laboratory (VDRL) test, at a titer of 1:32. Fluorescent treponemal antibody absorption test showed reactivity, whereas results of the HIV test were negative. On the 3rd day, analysis of cerebrospinal fluid through a lumbar puncture showed a positive VDRL result, at a titer of 1:8, and increased white blood cell count (22/mm^3^) and total protein (78.7 mg/dL). Since a positive VDRL result for the cerebrospinal fluid confirmed the diagnosis of neurosyphilis [4], the patient was administered intravenous penicillin G, 20 million units daily, for 2 weeks. Along with intravenous penicillin G, an oral antipsychotic regimen (blonanserin, 8 mg/day) was maintained throughout the treatment period. From the 7th day onward, the manic and psychotic symptoms improved dramatically, which was supported by his report of gaining insight into illness and significant reduction of the scores of psychiatric symptom rating scales (Table 1). By the time of discharge, on the 17th day, most psychiatric symptoms had disappeared. After 5 days of discharge, the patient visited our hospital, and neither mood symptoms nor psychotic symptoms were observed. He was administered a reduced dose of antipsychotic medication (blonanserin, 4 mg/day) for 2 weeks, which he later discontinued.

**Table 1:** Change in scores of psychiatric symptom rating scales after admission

	Admission	7th day	Discharge
YMRS	27	9	4
PANSS			
Total	56	38	32
Positive subscale	19	10	7
Negative subscale	10	8	7
General psychopathology subscale	27	20	18

*YMRS* Young Mania Rating Scale, *PANSS* Positive and Negative Syndrome Scale

## Discussion and conclusions

Although psychiatric symptoms are rarely encountered as the sole clinical manifestation of neurosyphilis nowadays, this case emphasizes that neurosyphilis should be considered as a potential differential diagnosis of neuropsychiatric syndromes in current psychiatric practice, even in HIV seronegative patients. Mitsonis et al. [6] reviewed the medical records of 81 hospitalized patients with neurosyphilis, from 1965 to 2005. During the period 1965–1984, only 27.4% of the patients with neurosyphilis exhibited psychiatric manifestations, whereas nearly 86% exhibited psychiatric symptoms during the period 1985–2005. In a previous retrospective review of neurosyphilis [5], the most common symptoms were personality change and memory impairment. Neurological abnormalities were also common, especially the absence of pupillary reaction to light and buccolingual masticatory movements. On the other hand, neurosyphilis presenting as paranoia, delusional thinking, and mood disorder has also been reported [7, 8]. Specific forms of hallucinations or schizophrenia-like symptoms as well as depressive and manic episodes were observed in patients with neurosyphilis [1, 8, 9]. Although there are no pathognomonic brain imaging findings that suggest neurosyphilis [4], several abnormal MRI findings, including generalized cerebral atrophy and foci of increased signal intensity on T2-weighted images, have been reported [10].

Interestingly, in the present case, prominent symptoms of psychotic mania were presented without any abnormalities in cognition, results of neurological examination, or brain MRI. Even though neurosyphilis may present with virtually any psychiatric symptom [1, 11], there has been only one case report of neurosyphilis presenting psychotic mania without any cognitive or neurological abnormalities [12]. Despite the patient fulfilling the diagnostic criteria for psychotic mania, some features of the case implied an organic cause of his manic-like episode with psychotic symptoms. Firstly, compared with over half the bipolar disorder patients having at least one family member with mood disorder [13], this case did not have any family history of psychiatric illness. Secondly, the patient showed the first manic-like episode in his late 40s, which is in contrast with the mean onset age of 30 years for bipolar disorder [14]. Moreover, given that patients with bipolar disorder typically show marked distractibility or hyperactivity during a manic episode, unimpaired cognition, particularly concentration, and absence of severe hyperactivity suggest an atypical feature of bipolar disorder, which emphasize the need to consider another disease as a differential diagnosis.

Penicillin has been considered as the treatment of choice for neurosyphilis [4]. The recommended regimen for neurosyphilis is 18–24 million units of intravenous aqueous penicillin G daily, either a continuous infusion or divided every 4 h, for 10–14 days [15]. In this case, the patient was administered blonanserin in addition to intravenous penicillin G, and after a few days, his psychiatric symptoms improved dramatically. To our knowledge, there has been no report as to the efficacy of blonanserin in the treatment of neurosyphilis. Blonanserin is a novel atypical antipsychotic drug which was approved for treatment of schizophrenia within the dose range of 8–24 mg/day in Japan and Korea [16, 17]. It has been shown to have potent antagonistic properties at dopamine D_2_ and serotonin 5-HT_2_A receptors, and is effective in alleviating positive and negative symptoms of schizophrenia [18, 19]. Although there are no specific guidelines to address psychiatric symptomatology, previous case series reported neurosyphilis-related psychotic symptoms to be treated successfully with typical or atypical antipsychotic drugs, concurrent with penicillin therapy [20]. The authors recommend that the lowest effective doses of antipsychotic agents could be utilized in severe cases requiring admission. Also, periodic attempts to reduce or withdraw these agents should be considered to minimize adverse events. Considering the rapid amelioration of psychotic mania-like symptoms, combination of blonanserin with penicillin may be more effective than penicillin monotherapy, suggesting a potential role of antipsychotic drugs in the treatment of psychiatric symptoms accompanied by neurosyphilis. Further, prognosis of the patient would be relatively good based on excellent treatment response, unimpaired cognition, and lack of neurological abnormalities.

This case report has several limitations. Although the possibility is thought to be low, it cannot be ruled out that the syphilis infection may have been caused by unrecognized manic symptoms of preexisting bipolar disorder. Moreover, given that the patient had experienced visual hallucinations, the MMSE may be insufficient for evaluation of cognitive function.

In conclusion, neurosyphilis remains a challenge in the field of clinical psychiatry because of its pleomorphic psychiatric symptomatology. Nevertheless, neurosyphilis is a treatable medical condition if it is accurately diagnosed early. The present case illustrates the difficulty in diagnosing neurosyphilis due to psychotic mania-like symptoms being the sole clinical manifestation. Thus, psychiatrists should keep in mind the need for a high index of suspicion and comprehensive laboratory work-up for possible syphilis when patients present with an abrupt onset of atypical psychiatric manifestation, especially in the absence of personal or family history of psychiatric illness. Also, this case suggests that combination of a low dose of blonanserin with antibiotic therapy may be an effective treatment option for psychotic mania-like symptoms associated with neurosyphilis.

## Abbreviations

HIV: human immunodeficiency virus
MMSE: Mini-Mental State examination
MRI: magnetic resonance imaging
VDRL: venereal disease research laboratory
